# Bayesian electron density determination from sparse and noisy single-molecule X-ray scattering images

**DOI:** 10.1126/sciadv.adp4425

**Published:** 2024-10-25

**Authors:** Steffen Schultze, Helmut Grubmüller

**Affiliations:** Max Planck Institute for Multidisciplinary Sciences, Am Fassberg 11, Göttingen, Germany.

## Abstract

Single molecule x-ray scattering experiments using free-electron lasers hold the potential to resolve biomolecular structures and structural ensembles. However, molecular electron density determination has so far not been achieved because of low photon counts, high noise levels, and low hit rates. Most approaches therefore focus on large specimen like entire viruses, which scatter sufficiently many photons to allow orientation determination of each image. Small specimens like proteins, however, scatter too few photons for the molecular orientations to be determined. Here, we present a rigorous Bayesian approach to overcome these limitations, additionally taking into account intensity fluctuations, beam polarization, irregular detector shapes, incoherent scattering, and background scattering. We demonstrate using synthetic scattering images that electron density determination of small proteins is possible in this extreme high noise Poisson regime. Tests on published virus data achieved the detector-limited resolution of 9 nm, using only 0.01% of the available photons per image.

## Teaser

Electron densities can be determined from noisy images without orientation determination.

## INTRODUCTION

**Fig. 1.**
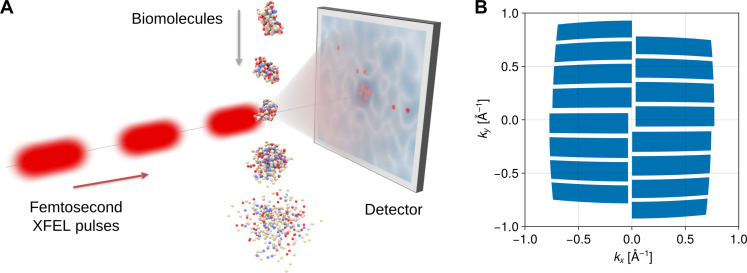
Single-molecule scattering experiment. (**A**) Experimental setup for single molecule x-ray scattering [image reproduced from von Ardenne *et al.* (*17*)]. (**B**) Irregular detector shape used for our simulated scattering experiments, modeled after the detector used at the European XFEL (*60*). Note that the apparent “curvature” does not reflect the actual detector geometry but is instead an artifact of the projection onto the Ewald sphere.

Ultrashort pulse x-ray scattering experiments using x-ray free-electron lasers (XFELs) offer the possibility to take “snapshots” of biomolecular structures with subnanometer spatial and femtoseconds time resolution (*1*–*4*). In these “diffraction before destruction” experiments (*5*) (Fig. 1A), a stream of sample particles is hit by a series of high intensity, ultrashort (femtoseconds) x-ray pulses; and for each pulse, the scattered photons are recorded as a scattering image. Crucially, the pulses are so short that scattering outruns sample destruction (*6*).

Currently, most of these experiments focus on nanocrystals. These serial femtosecond crystallography experiments have provided both static (*7*, *8*) and time-resolved structures at resolutions better than 3 Å (*9*–*11*). Despite these successes, the need to grow sufficiently well-ordered crystals and the inevitable ensemble averaging pose severe limitations (*11*).

The “holy grail” is therefore to perform x-ray scattering experiments on single noncrystalline particles or even single molecules such as proteins (*3*, *6*). Although the high repetition rates of current XFELs of up to 27 kHz (*12*) allow the collection of millions of such images even at rather low hit rates (*13*), substantial challenges remain, such as low photon counts, unknown sample orientation, and low signal-to-noise ratios. These have so far limited such experiments to relatively large particles such as viruses at moderate resolutions of ca. 10 nm (*14*–*16*). Here, we address these challenges using a rigorous Bayesian approach, and demonstrate that de novo electron density determination should be possible also for single molecules.

The first challenge is that, because of the small molecular size, the number of scattered photons is typically very low (*6*). For single proteins, and despite the high photon flux provided by the XFEL, only ten to several hundred recorded photons per scattering image are expected (*17*). In this extreme Poisson regime, each scattering image thus does not reveal the full scattering intensity distribution (light blue color on the detector, Fig. 1A), but rather consists of only a few discrete photon positions (red dots), distributed according to the unknown scattering intensity.

The second challenge is that, for each hit, the sample orientation is different, random, and unknown, preventing a naive approach based on averaging many images. Several new methods have been developed over the past 20 years to overcome this challenge. Most methods aim to determine the molecular orientation from the positions of the scattered photons separately for each image. Subsequently, the properly oriented images are assembled in Fourier space into a full three-dimensional scattering intensity (*18*–*25*), from which the electron density is derived using established phase retrieval methods (*26*–*28*). These orientation determination approaches, including the expansion-maximization-compression (EMC) algorithm (*13*, *25*, *29*) and manifold embedding algorithms such as diffusion map (*30*–*33*), typically require 10^2^ to 10^4^ coherently scattered photons per image to determine the sample orientation with sufficient accuracy. Moreover, they are rather sensitive to noise, precluding their application to single molecules. For realistic noise levels and photon counts, the information content per scattering image is far too low for successful orientation determination of individual images (*20*).

To circumvent this problem, it has been proposed to extract only orientation-invariant quantities from the scattering images, in particular correlations (*17*, *34*–*41*). Notably, using three-photon correlations, it has been shown that density determination should be possible from as few as three recorded photons per image (*17*, *42*) as the ultimate limit. However, by neglecting higher correlations, much of the scattering information is discarded (*17*).

The third challenge is posed by several additional sources of experimental noise and uncertainties, mainly due to incoherent scattering, background scattering, and beam intensity fluctuations, as well as incomplete and irregular coverage of the scattering solid angle by the detector (Fig. 1B) (*2*, *17*, *19*, *20*). These noise sources are particularly prohibitive at the single-particle level, and the usual subtraction of an estimated background noise level and fortuitous error cancellation through averaging fail in this extreme Poisson regime with noise levels of up to 90% (*13*, *43*, *44*). Because of the lack of a combined, systematic treatment of all three sources of uncertainty, de novo electron density determination of single proteins has so far been out of reach.

The Bayesian method we developed and assessed here approaches the problem from a different angle. Rather than attempting to orient each individual scattering image, the Bayesian posterior probability given the whole set of images (typically thousands to millions) is considered and either sampled or maximized. The posterior is computed by marginalizing over all possible orientations for each image, thereby avoiding the need to determine or estimate the orientation of each single image. As a further important advantage, this Bayesian approach allows for a systematic inclusion of noise in terms of a physics-based forward model of the scattering experiment and its uncertainties. Further, and in contrast to correlation-based methods, the full information content of all scattering images is used, thus reducing the required number of images to achieve a particular resolution. Last, posterior sampling provides error bounds and uncertainty estimates for the obtained electron density.

We tested our approach both on noisy synthetic scattering images and on downsampled experimental images. Even for a small single protein (crambin), resolutions of 8 to 10.4 Å were achieved under realistic conditions, and up to 4.2 Å under noise-free conditions. As a test using experimental images, we successfully recovered the electron density of the coliphage PR772 (*45*) at 9-nm detector limited resolution using only 0.01% of the recorded photons per image.

## RESULTS

### Bayesian inference of single-molecule x-ray scattering

We first summarize the Bayesian formalism and our approach. For each scattering event *j* = 1…*N*, the positions of the *n_j_* scattered photons are recorded on the detector as a scattering image and are converted into scattering vectors $k_{1}^{\left(j\right)},\ldots ,k_{n_{j}}^{\left(j\right)}$. For each possible electron density function ρ, a Bayesian posterior probability is calculated given the set of scattering images $ℐ={\left\{k_{1}^{\left(j\right)},\ldots ,k_{n_{j}}^{\left(j\right)}\right\}}_{j=1\ldots N}$

P(ρ∣ℐ)∝P(ℐ∣ρ)P(ρ) (1)

from which the most probable electron density as well as its uncertainty is derived.

The likelihood function *P*(ℐ∣ρ) contains an appropriate physical forward model of the scattering process, including noise, intensity fluctuations, polarization, and irregular detector shapes (see Materials and Methods). Because each image is an independent event, the likelihood decomposes into a product of the likelihoods of each single image *j*

$$P\left(ℐ|\rho \right)=\prod_{j=1}^{N}P\left(k_{1}^{\left(j\right)},\ldots ,k_{n_{j}}^{\left(j\right)}|\rho \right)$$ (2)

In the absence of information on the orientation of the sample molecule during each scattering event, the single-image likelihood function is given by marginalizing, i.e., as an average over all possible orientations **R** of the corresponding conditionalized probability of the scattering image *j*

$$P\left(k_{1}^{\left(j\right)},\ldots ,k_{n_{j}}^{\left(j\right)}|\rho \right)=\int_{\text{SO}\left(3\right)}P\;(k_{1}^{\left(j\right)},\ldots ,k_{n_{j}}^{\left(j\right)}|\rho ,R)\;dR$$ (3)

Here, SO(3) denotes the set of all three-dimensional rotation matrices. The latter was calculated from the forward scattering model as described in Materials and Methods. To maximize or sample from the Bayesian posterior, a combined Markov chain Monte Carlo (MCMC) simulated annealing approach was used.

As a physics-motivated real-space representation of the electron density ρ, we chose a sum of Gaussian functions, with size and number depending on the target resolution. This choice serves both to minimize the number of required degrees of freedom and as a means of regularization. While one may think of these Gaussians as representation of, for example, an atom, an amino acid, or a larger domain of the sample, their purpose is only to represent electron densities, not structural entities. Notably, by using such a representation for the electron density as opposed to its Fourier transform, our approach circumvents the phasing problem.

For a typical target protein consisting of up to several hundred amino acids, the number of required degrees of freedom remains nevertheless large and poses a formidable sampling challenge. To achieve sufficient sampling, we applied a hierarchical simulated annealing approach as described in Materials and Methods. In brief, starting at very low resolution and correspondingly few Gaussian functions, the electron densities were sampled in multiple hierarchical stages of increasing resolution; and in each of these stages, the previous electron density of maximal posterior probability was used as a proposal density for the MCMC steps.

### Electron density reconstruction from noise-free images

**Fig. 2.**
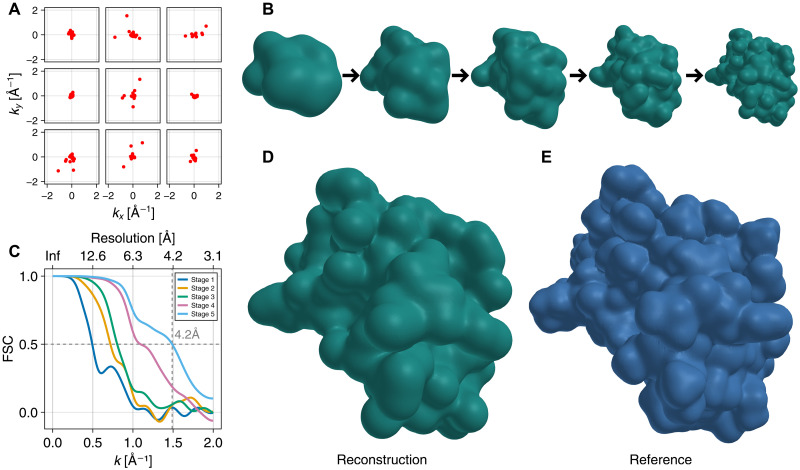
Electron density determination from noise-free images. (**A**) Sample synthetic noise-free images, containing only coherent signal photons (red dots). (**B**) Hierarchical stages of retrieved electron densities. (**C**) Fourier shell correlation between reconstructed and ground-truth reference density. (**D**) Reconstructed electron density. (**E**) Reference electron density.

We first tested our method on synthetic noise-free images, using the same 46–amino acid protein crambin (*46*) that was used for the assessment of previous correlation-based methods (*17*). Because our Bayesian approach uses all available information, we expect it to require fewer scattering images to achieve the same resolution. To test this expectation, a total of 10^8^ noise-free synthetic scattering images were generated, containing a realistic average number of 15 photons each (*17*, *47*). As described in Materials and Methods, for each image, a random molecular orientation was chosen, and for each orientation, the number of photons per image was drawn from a Poisson distribution. Here, an intensity of 10^12^ photons per pulse was assumed with a beam diameter of 1 μm (*43*). Figure 2A shows several of these images as an example.

From these images, the electron density was determined in five hierarchical stages (Fig. 2b), increasing the number of Gaussian functions representing the electron density ρ by a factor of two in each stage. For the final stage, 184 Gaussian functions were used, which is four times the number of amino acids. For more details, see table S1. A similar Fourier shell correlation resolution (*48*) of 4.2 Å (Fig. 2C) was obtained as in the previous study of our group (*17*) using only half the number of scattered photons. Here, the Fourier shell correlations were used to compare the reconstructed electron density map (Fig. 2D) with the ground-truth density (Fig. 2E) that was used to generate the synthetic scattering images. As a further measure of quality, the optimal transport plan between the reconstructed and reference electron densities was computed using a standard algorithm (*49*), obtaining an earth mover’s distance of 1.45 Å.

### Density determination from noisy images

**Fig. 3.**
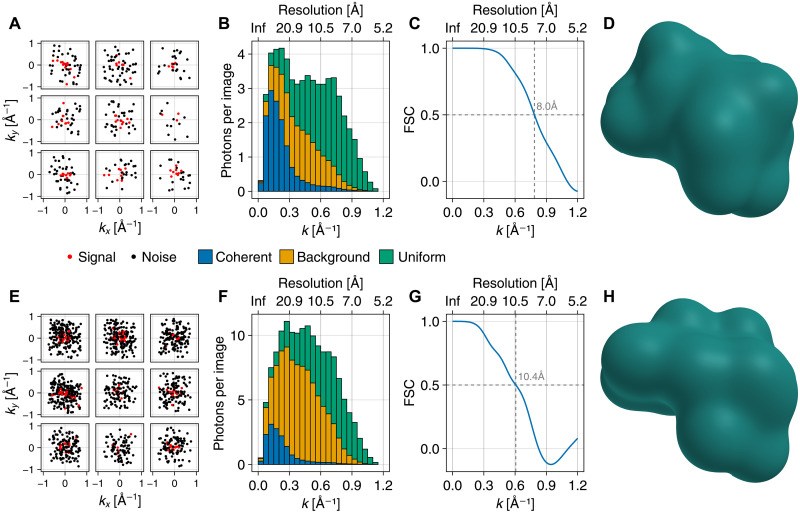
Electron density determination from noisy images. Electron density determination at [(A) to (D)] a noise level of 60% and [(E) to (H)] of 90%. (**A** and **E**) Sample synthetic noisy images, showing coherent signal photons (red) and noise photons (black). (**B** and **F**) Radial distribution of photons from coherent scattering, background, and incoherent (uniform) noise (stacked histogram). (**C** and **G**) Fourier shell correlations between reconstructed and ground-truth reference densities show the achieved resolutions of 8 Å and 10.4 Å. (**D** and **H**) Reconstructed electron densities.

To assess our method also in the presence of realistic noise sources, we tested it on synthetic noisy scattering images for the same protein crambin (*46*). Because estimates for the experimentally achievable noise level vary and depend on the exact experimental setup (*13*, *43*, *44*), two different noise levels were considered: One million synthetic images were generated at a noise level of 75% (Fig. 3, A to D), and three million at a noise level of 90% (Fig. 3, E to H), which both are within experimental reach (*13*, *43*, *44*). Figure 3 (A and E) shows representative examples of these images. Here, the same average number of 15 coherently scattered signal photons (red) per image as above was assumed. Only these contain structural information but are indistinguishable from the noise photons (black). In addition, a total of on average 44 (Fig. 3A) or 137 (Fig. 3E) photons per images of all detected photons were assumed to be incoherent. As shown in Fig. 3 (B and F), these were assumed to be from background scattering on carrier gas or solvent molecules (orange, described here by a normal distribution with SD 0.35 Å^−1^), as well as from incoherent scattering (green, described by a uniform distribution). For more details, see Materials and Methods and table S1.

Despite these low signal-to-noise ratios, Fig. 3 (C and G) shows that structural information is recovered at a conservative Fourier shell correlation resolution estimate of 8 Å in the case of 75% noise and 10.4 Å in the case of 90% noise. Here, representations consisting of 12 and 23 Gaussians were used. Note that the smaller number of Gaussians was adapted to the expected lower achievable resolution of the obtained electron densities compared to the noise-free images. Figure 3 (D and F) shows the obtained electron densities that represent the overall shape of the molecule at the two resolution levels.

### Application to experimental data

**Fig. 4.**
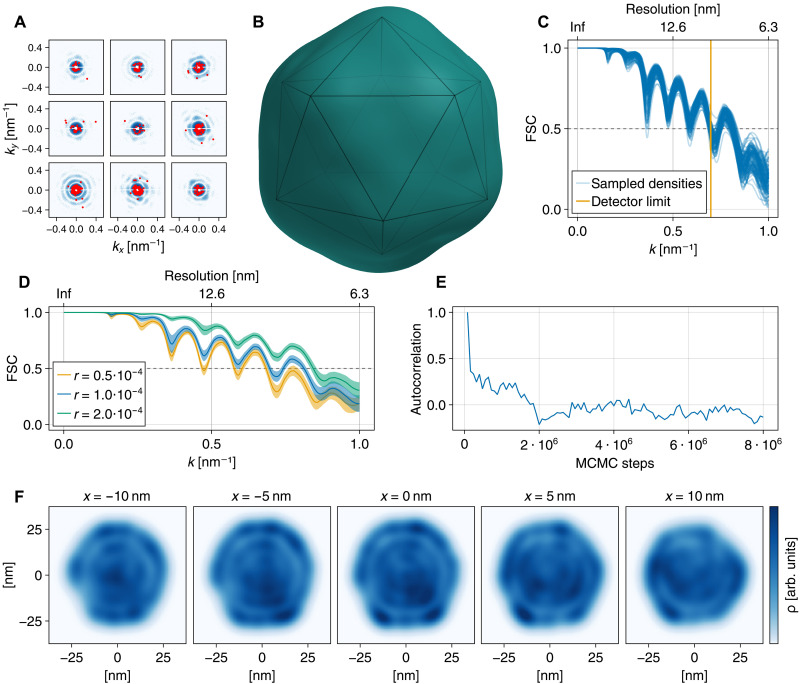
Electron density determination of the coliphage PR772. (**A**) Sample downsampled images (red dots) and corresponding original experimental images (blue, log scaled) at a downsampling factor of *r* = 10^−4^. (**B**) Average density obtained from MCMC-sampling. Perfect icosahedron with side length 30 nm for reference. (**C**) Fourier shell correlations between 100 randomly selected sampled electron densities and the average density. (**D**) Average Fourier shell correlations (with SD) obtained for three downsampling ratios *r*. (**E**) Autocorrelation of electron densities as a function of MCMC steps. (**F**) Slices through the average electron density obtained for *r* = 10^−4^ on the *yz* plane for various values of *x*.

Having assessed our method using synthetic data for which the “ground truth” is known, we next tested it on published experimental data for the icosahedral coliphage PR772 (*45*). Because this virus is much larger than the protein molecule considered above, on average about 400,000 photons per scattering image were recorded. Notably, only photons up to *k* = 0.69 nm^−1^ were recorded, which limits the resolution to 9 nm. For a fair assessment, and to mimic the more challenging low photon counts expected for single-molecule scattering experiments, we downsampled the original images by a factor of *r* = 10^−4^ using rejection sampling to obtain images with an average of 40 photons per image (Fig. 4A). A dataset consisting of 2 ⋅ 10^5^ such images was emulated by randomly downsampling the same images multiple times. Because the intensity distribution of the incoming beam is unknown due to the applied hit selection, intensity fluctuations were taken into account via normalization as described in Materials and Methods.

The virus electron density was described by *n* = 400 Gaussians functions of width σ = 3 nm, adapted to the resolution set by the experimental data as described in Materials and Methods. To better represent the virus electron density at this scale, both the positions **y***_i_* and the heights *h_i_* of the Gaussian functions were considered unknown and determined during the annealing. After the annealing procedure, the density was further sampled for 8 ⋅ 10^6^ MCMC steps. As shown in Fig. 4E, the autocorrelation between these MCMC samples decreases to zero after about 2,000,000 steps, suggesting that a sufficient number of MCMC samples are largely uncorrelated. The thus obtained sample of electron densities was rotationally aligned and averaged. The resulting density (Fig. 4B) resembles the expected icosahedral structure of the virus. As shown in Fig. 4F, even the internal structure consisting of multiple concentric shells was resolved. An animated version of Fig. 4B is provided as movie S1.

To obtain an error estimate, the Fourier shell correlations of a randomly selected small subset of these sampled electron densities relative to the averaged density were computed (Fig. 4C). As can be seen, the 9-nm resolution limit imposed by the detector geometry was largely achieved. In contrast to Hosseinizadeh *et al.* (*29*), icosahedral symmetry was not imposed, and therefore, the electron density determined here deviates from a perfect icosahedral symmetry (as is also obvious from the slices shown in Fig. 4F), which has also been observed for this dataset using other methods (*40*, *50*).

Last, we asked how the obtained resolution depends on the number of photons per image, here governed by the downsampling ratio. To that end, further runs were performed at downsampling ratios *r* = 5 ⋅ 10^−5^ and *r* = 2 ⋅ 10^−4^, that is, on average, 20 and 80 photons per image, respectively. The electron densities obtained from these runs are shown in fig. S1. As shown in Fig. 4D, the Fourier shell correlation does increase with increasing number of photons per image, as one might expect. Note, however, that this does necessarily imply a further increase in resolution, because, as mentioned above, only photons up to *k* = 0.7 nm^−1^ were recorded in the experiment. The larger Fourier shell correlation values at *k* > 0.7 nm^−1^ likely result rather from an increasing overdetermination of the positions of the Gaussians. We therefore also investigated how the determined density depends on the number of used Gaussians (see fig. S2) and found that for 200 and 300 Gaussians, respectively, granularity artifacts are seen in the electron density, in particular for 200 Gaussians, indicating that this number is insufficient for an accurate representation of the electron density.

## DISCUSSION

We have demonstrated electron density determination from highly noisy and sparse single-particle x-ray scattering images using a rigorous Bayesian approach. Uncertainties such as unknown sample orientation, beam intensity fluctuations, polarization, irregular detector shapes, Poisson noise due to the typically very few recorded photons per image, and noise from incoherent and background scattering have been taken into account using a realistic physics-based forward model. This model can be adapted to specific experimental conditions and can be generalized to include other noise sources such as detector noise as well.

Our simulated scattering experiments demonstrate that electron densities can be reliably determined even in this high noise regime well beyond 1-nm resolution. There is no fundamental limit to achieving even higher resolutions, given sufficient numbers of scattering images. In contrast, for approaches based on orientation determination, the resolution is limited by the number of photons per image (*20*). While for approaches based on orientation determination the resolution is limited by the number of photons per image (*20*), there is no fundamental limit for our Bayesian approach to achieving even higher resolutions, given sufficient numbers of scattering images.

Because our approach uses all available information, fewer scattering images and fewer photons per image are required to achieve the same resolution than by previous approaches, in particular compared to correlation methods (*17*). For the coliphage test case, 10^4^ times downsampled images sufficed to recover the icosahedral structure at the detector-limited resolution of 9 nm. Ten to 100 times fewer photons per image were required than what was so far considered the “low signal limit” (*50*). For this comparison, note that our photon counts refer to entire images, whereas Ayyer *et al.* (*50*) report photon counts excluding the central speckle that contains most of the photons.

Although there is no fundamental resolution limit to our approach, Bayesian sampling in high-dimensional search spaces generally poses computational challenges. The problem-adapted hierarchical sampling method presented here alleviated this technical limitation markedly and allowed the optimization of electron density representations with up to 1200 degrees of freedom. Nevertheless, for increasing sample size to resolution ratios, the main bottleneck of our approach is the computational effort, both due to the required sampling and the large number of scattering images. For example, the final stage in the noise-free test scenario took about 1500 GPU-hours of parallel computation time (Nvidia RTX 3090) and the MCMC sampling for the coliphage about 1600 GPU-hours (Nvidia RTX 4070). Although a brute-force approach is possible due to inherently parallel computations, improved optimization and sampling methods will be helpful to address this issue (*51*), as may the use of prior structural information from, for example, structure databases, AlphaFold (*52*), or molecular dynamics force fields.

This computational bottleneck does not preclude the application to larger specimen, like the coliphage studied here. Rather, the computational effort increases with the complexity of the used electron density representation. Whereas we have, under ideal conditions, demonstrated that a resolution of 4.2 Å can be achieved for a small soluble protein, for larger complexes such as the ribosome, substantial computational resources would be required to achieve resolutions better than about 3 nm.

Whereas our results demonstrate that our Bayesian approach should enable structure determination from noisy single-molecule x-ray scattering images, we have so far only assessed its performance and accuracy on synthetic scattering images or on preprocessed images from diffraction experiments on much larger virus specimen. Because the approach only rests on a physical forward model of the experiment, further sources of noise of experimental uncertainties can be readily implemented in a systematic way. Although the forward model presented in this work turned out to be sufficiently accurate to enable successful reconstruction of the PR772 virus, future calibration and improvements will be beneficial, such as more complex detector models, the effect of a possible solvation shell around the molecule, structural heterogeneity, or the identification of hits versus misses.

Of note, at intensities higher than 10^19^ W/cm^2^, as required for smaller particles or single molecules, ultrafast ionization distorts the electron density already during the few femtoseconds exposure (*53*) and, hence, presents a further challenge for its reconstruction. Although simulations of these electron dynamics have been reported (*54*), more research will be required to include these within our Bayesian framework.

From a more general perspective, our fully Bayesian approach should also be transferable to other imaging methods with high noise levels. In particular, single-molecule cryogenic electron microscopy (cryo-EM) shares many similarities, in particular unknown and random orientations of the sample molecule. As a result, also established analysis methods, such as RELION for cryo-EM (*55*) and EMC for XFEL imaging (*25*), both rest on the same mathematical technique (expectation maximization). It will therefore be very interesting to see if our approach is able to also extract more information for these experiments.

## MATERIALS AND METHODS

### Noise-free forward model

In the experiments, single sample molecules enter a pulsed femtosecond XFEL beam, and for each pulse, the positions of the scattered photons are recorded on the detector as a scattering image. Each location on the detector corresponds to a specific scattering vector **k** = **k**_i_−**k**_s_ on the Ewald sphere *E* in Fourier space, where **k**_i_ is the incident wave vector and **k**_s_ the wave vector after scattering. Each scattering image is, therefore, given by a list of scattering vectors **k**_1_,…, **k***_l_*. Their probability distribution is given by three-dimensional intensity function *I*_ρ_(**Rk**) = ∥ ℱ {ρ}(**Rk**)∥^2^, which for coherent scattering is given by the Fourier transform of the electron density ρ. Here, **R** ∈ SO(3) is a rotation matrix describing the orientation of the molecule.

The likelihood that an image **k**_1_,…, **k***_l_* is observed for a given electron density ρ is obtained by averaging the conditional likelihood over all possible orientations **R**. This conditional likelihood is given by the product of a Poisson distribution for the number of photons *l* and, because the photons are conditionally independent given **R**, a product of the intensity function evaluated at the scattering vectors of the scattered photons

$$P\left(k_{1},\ldots ,k_{l}|\rho \right)\propto \int_{\text{SO}\left(3\right)}dRI_{0}^{l}\text{exp}\left[-I_{0}\int_{E}I_{\rho }\left(\text{Rk}\right)\;dk\right]\prod_{i=1}^{l}I_{\rho }\left({\text{Rk}}_{i}\right)$$ (4)

Here, *I*_0_ represents the incoming beam intensity. Note that here and subsequently normalization factors and constants such as the electron radius are omitted. Instead, they are absorbed into the value of *I*_0_, which is chosen at the end such that the correct photon counts are obtained.

This likelihood, given by Eqs. 2 and 4, represents the complete noise-free forward model, which forms the basis for the subsequent inclusion of error models.

### Incoherent and background scattering

In addition to the coherent photons, also incoherently scattered photons from, for example, Compton scattering and Auger decay, are observed. They represent up to 90% of the total scattered photons but are distributed uniformly on the Ewald sphere. They therefore spread over a much larger solid angle than the coherent photons, such that the effective amount of noise due to this incoherent scattering is smaller. For this reason, the noise due to incoherent scattering is larger for increasing resolutions, whereas at lower resolutions of about 10 nm that have been demonstrated for viruses, it can be neglected.

A second source of noise is scattering from other molecules, such as water molecules attached to the sample in aerosol delivery (*56*), bulk water for liquid beam (*57*) or sheet (*58*) delivery, or remaining gas molecules in the beam volume. These molecules scatter both coherently and incoherently, but, because of the random positions and orientations of these particles, incoherent summation to *I*_ρ_ is a good approximation.

Neglecting beam polarization for a moment, the distribution of the photons from incoherent and background scattering is radially symmetric. For simplicity, here, a uniform distribution on the Ewald sphere is assumed for the incoherently scattered photons and a Gaussian distribution centered at the origin of reciprocal space for the background scattering. Other radial distributions, for example, from measurements, can of course be readily implemented.

To include incoherent and background scattering within the likelihood function, their distributions are added to the intensity function, replacing *I*_ρ_ by

$$I_{n}\left(k,\rho \right)=I_{\rho }\left(k\right)+I_{b}\left(k\right)+I_{u}$$ (5)

in Eq. 4. Here, the constant *I*_u_ describes the contribution from uniform incoherent scattering, and

$$I_{b}\left(k\right)=\frac{C_{b}}{2{\pi \sigma }^{2}}\text{exp}\left(-\frac{k^{2}}{2\sigma^{2}}\right)$$ (6)

is the Gaussian distribution describing background scattering.

### Polarization

To additionally include the linear polarization of the XFEL beam, the scattering intensity is changed by a factor *f*_p_(**k**) = cos^2^θ + cos^2^ϕsin^2^θ = 1−(*k_y_*λ/2π)^2^, where θ is the scattering angle and ϕ the azimuthal angle relative to the direction of polarization (*59*). As a consequence, for each scattering vector **k**, the expected number of photons from coherent and Compton scattering is reduced by *f*_p_(**k**). In contrast, the distribution of photons arising from Auger decay is unaffected. For our forward model, we assume therefore that the Gaussian noise *I*_b_ is multiplied by this factor while the uniform noise is not.

Accordingly, *I*_n_ is replaced by

$$I_{\text{np}}\left(R,k,\rho \right)=f_{p}\left(k\right)\left[I_{\rho }\left(\text{Rk}\right)+I_{b}\left(k\right)\right]+I_{u}$$ (7)

which now also depends on the molecular orientation. As result, rotating the molecule around the beam axis does no longer correspond directly to rotating the scattering images because the polarization orientation is stationary. The likelihood becomes

$$P\left(k_{1},\ldots ,k_{l}|\rho \right)\propto \int_{\text{SO}\left(3\right)}dRI_{0}^{l}\text{exp}\left[-I_{0}\int_{E}dkI_{\text{np}}\left(R,k,\rho \right)\right]\prod_{i=1}^{l}I_{\text{np}}\left(R,k_{i},\rho \right)$$ (8)

### Irregular detector shape

Most x-ray detectors have irregular shapes. For example, the detector used at the European XFEL (*60*) is composed of 16 separate modules arranged as shown in Fig. 1B. In the forward model, the shape of the detector is encoded in the detection probability *p*_d_(**k**) that a photon with scattering vector **k** is registered by the detector. This formalism allows for the inclusion of any detector shape and can also be used to include individual detection probabilities per pixel.

The resulting likelihood function is a straightforward extension similar to the above polarization, the only difference being that here, all photons are affected. As a consequence, the factors *p*_d_(**k***_i_*) in the product over *i* factor out and can be omitted because they do not depend on the images

$$\begin{array}{c} P\left(k_{1},\ldots ,k_{l}\;|\rho \right)\propto \int_{\text{SO}\left(3\right)}dR\;I_{0}^{l}\text{exp}\left[-I_{0}\int_{E}dk\;p_{d}\left(k\right)I_{\text{np}}\left(R,k,\rho \right)\right]\prod_{i=1}^{l}p_{d}\left(k_{i}\right)I_{\text{np}}\left(R,k_{i},\rho \right)\\ \propto \int_{\text{SO}\left(3\right)}dR\;I_{0}^{l}\text{exp}\left[-I_{0}\int_{E}dk\;p_{d}\left(k\right)I_{\text{np}}\left(R,k,\rho \right)\right]\prod_{i=1}^{l}I_{\text{np}}\left(R,k_{i},\rho \right) \end{array}$$ (9)

### Intensity fluctuations

Fluctuations of the incoming beam intensity *I*_0_ are described by a γ distribution *I*_0_ ∼ 〈*I*_0_〉Γ(α, β), where 〈*I*_0_〉 is the average intensity (*61*–*63*). The shape and rate parameters α and β depend on the specific free-electron laser. For the forward model, α = β = 4 was assumed, which has been determined for an XFEL operating at 32-nm wavelength (*63*).

To include these fluctuations within the likelihood function, an additional integral over *I*_0_ weighted by the probability density of the gamma distribution is required

$$\begin{array}{c} P\left(k_{1},\ldots ,k_{l}|\rho \right)\propto \int_{0}^{\infty }dI_{0}\;I_{0}^{\alpha -1}\text{exp}\left(-\frac{\beta I_{0}}{\langle I_{0}\rangle }\right)\int_{\text{SO}\left(3\right)}\\ dRI_{0}^{l}\text{exp}\left[-I_{0}\int_{E}dkp_{d}\left(k\right)I_{\text{np}}\left(R,k,\rho \right)\right]\prod_{i=1}^{l}I_{\text{np}}\left(R,k_{i},\rho \right) \end{array}$$ (10)

Conveniently, this integral can be carried out analytically, and the likelihood reads

$$\begin{array}{c} P\left(k_{1},\ldots ,k_{l}|\rho \right)\propto \int_{\text{SO}\left(3\right)}dR\Gamma \left(l+\alpha \right){\left[\frac{\beta }{\langle I_{0}\rangle }+\int_{E}dk\;p_{d}\left(k\right)I_{\text{np}}(R,k,\rho )\right]}^{-l-\alpha }\\ \prod_{i=1}^{l}I_{\text{np}}\left(R,k_{i},\rho \right) \end{array}$$ (11)

The likelihood function in Eq. 11 represents the so far complete forward model, including incoherently scattered photons, background scattering, beam polarization, the irregular detector shape, and intensity fluctuations. Accordingly, here, it was used for the tests on noisy synthetic data.

Note that Eq. 11 assumes knowledge of the probability distribution of *I*_0_. Such knowledge is not always given, however. An example is the coliphage dataset considered here. For this dataset, the images were preselected such that only images classified as hits were retained, and because the hit selection is affected by the number of photons per image, the beam intensity distribution distribution for the selected images is not well defined and best regarded as unknown. To adapt the likelihood function to this situation, intensity fluctuations were treated by considering the probabilities conditionalized on the number of photons *l*, replacing *P*(**k**_1_,…, **k***_l_* | ρ) with

$$\begin{array}{c} P\left(k_{1},\ldots ,k_{l}|\rho ,l\right)\propto \int_{\text{SO}\left(3\right)}dR{\left[\int_{E}dk\;p_{d}\left(k\right)I_{\text{np}}\left(R,k,\rho \right)\right]}^{-l}\\ \prod_{i=1}^{l}I_{\text{np}}\left(R,k_{i},\rho \right) \end{array}$$ (12)

in Eq. 2. As this form is independent of *I*_0_, the intensity does not have to be integrated as a nuisance parameter. The importance of the likelihood function is illustrated in fig. S3, showing a virus electron density determined using the “wrong” likelihood from Eq. 11.

### Structure representation

Electron density functions of the reference structures were described by a sum of *m* Gaussian beads with positions **y***_i_*, heights *h_i_* and SDs σ*_i_*

$$\rho \left(r\right)=\sum_{i=1}^{m}\frac{h_{i}}{{\left(\sigma_{i}\sqrt{2\pi }\right)}^{3}}\text{exp}\left(-\frac{1}{2\sigma_{i}^{2}}∥r-y_{i}{∥}^{2}\right)$$ (13)

Electron density functions of the determined electron densities were described similarly, with one common SD σ = σ*_i_* and one common height *h* = *h_i_* for the tests on crambin but independent heights *h_i_* for the test on the coliphage. These were treated as unknowns and determined together with the positions **y***_i_*.

The number of Gaussian functions *m* has been chosen heuristically. The main criterion was that it needs to high enough to accurately represent the electron density at the target resolution. For the tests on crambin, we chose *m* as multiples of the number of amino acids. As an estimate, the maximum achievable resolution using *m* Gaussians is obtained by the condition that the volume of the sum of m Gaussians must equal the known volume *V* of the molecular electron density, resulting in an estimated width $\sigma =\sqrt[{3}]{3/\left(4\pi \right)\cdot V/m}$ of each Gaussian. Empirically, the maximal achievable Fourier shell correlation resolution (using the threshold of 0.5 as described later) corresponds to about three times this width. Last, estimating the volume as *V* ≈ 5 Å^3^ ⋅ *N*_heavy_, where *N*_heavy_ is the number of heavy atoms of the molecule, an estimate for the achievable resolution of $3\sqrt[{3}]{3/\left(4\pi \right)\cdot 5}\cdot \sqrt[{3}]{N_{\text{heavy}}/m}$ Å $\approx 3.2\sqrt[{3}]{N_{\text{heavy}}/m}$ Å is obtained. From these heuristics we expect to achieve a resolution of 9.5 Å for crambin using 12 Gaussians, and 3.8 Å using 184 Gaussians, which is very close to the results of our tests. For the coliphage test case, the volume was estimated as *V* = 4π/3 ⋅ (25 nm)^3^. Here, the above estimate suggests that *m* = 400 Gaussians should be required to approach the detector-limited resolution of 9 nm, which also agrees very well with our findings that a sufficiently well-defined nongranular electron density is obtained only for this number of Gaussians.

### Simulated scattering experiments

The forward model described in above was simulated by the following procedure. To generate one scattering image from an electron density ρ,

1. Draw the intensity *I*_0_ ∼ 〈*I*_0_〉Γ(α, β).

2. Draw the orientation $R∼U\left[\text{SO}\left(3\right)\right]$ from a uniform distribution on the rotation group.

3. Draw $\bar{l}∼\text{Pois}\left[I_{0}4\pi {\left(2\pi /\lambda \right)}^{2}I\left(R,0,\rho \right)\right]$, with the intensity function *I* = *I*_ρ_ for noise-free images or *I* = *I*_np_ from Eq. 7 for noisy images.

4. Draw photon positions $k_{1},\ldots ,k_{\bar{l}}$ uniformly distributed on the Ewald sphere, and accept each with probability *p*_d_(**k***_i_*)*I*_np_(**R**, **k***_i_*, ρ)/*I*_np_(**R**, **0**, ρ).

Note that this procedure works correctly because the intensity function *I*(**R**, **k**, ρ) is always maximal at **k** = **0**. To generate the noise-free scattering images, *I*_0_ was instead set as a constant *I*_0_ = 〈*I*_0_〉, and the detector geometry was given by *p*_d_(**k**) = 1.

### Computation of likelihoods

The integral over the orientation **R** in Eqs. 4, 11, and 12 was approximated by averaging over a discrete set of typically *n* ≈ 10^3^ to *n* ≈ 10^5^ rotations **R***_i_* with weights *w_i_*. For example, in the noise-free case Eq. 4 was approximated as

$$\begin{array}{c} P\left(k_{1},\ldots ,k_{l}|\rho \right)\approx \sum_{i=1}^{n}w_{i}I_{0}^{l}\text{exp}\left[-N\int_{E}I_{\rho }\left(R_{i}k\right)\;dk\right]\\ \prod_{j=1}^{l}I_{\rho }\left(R_{i}k_{j}\right) \end{array}$$ (14)

The rotations **R***_i_* and their weights *s_i_* were constructed by combining a Lebedev quadrature rule on *S*^2^ (*64*) with a uniform quadrature rule on *S*^1^, as described by Gräf and Potts (*65*). To further increase the computational efficiency, the photon positions **k***_i_* were discretized. For a more detailed explanation and further implementation details, see the Supplementary Text.

### Monte Carlo–simulated annealing

Optimization and posterior sampling was performed via MCMC-simulated annealing (*66*). An exponential temperature schedule *T*(*t*) = *T*_0_ exp (−*t* ln 2 / *t*_1/2_) was used, with *T*_0_ and *t*_1/2_ as listed in table S1. The sampling challenge due to the high number of degrees of freedom at high resolutions was alleviated by determining the electron density in multiple hierarchical stages with an increasing number of Gaussian beads. In each stage, the density from the previous stage was used as a proposal density, markedly increasing the sampling performance. To define this proposal density, let **y**_1_,…, **y***_n_* be the positions of the Gaussian functions from the previous stage and **z**_1_, …, **z***_m_* those of the current stage. Then, the proposal density was, up to normalization, given by

$$\begin{array}{c} g\left({z\prime }_{1},\ldots ,{z\prime }_{m}|z_{1},\ldots ,z_{m}\right)\propto \prod_{i=1}^{m}\text{exp}\left(-\frac{∥{z\prime }_{i}-z_{i}{∥}^{2}}{2d^{2}}\right)\\ \text{exp}\;\left(-\frac{∥{z\prime }_{i}-y_{i\text{mod}n}{∥}^{2}}{2w^{2}}\right) \end{array}$$ (15)

where *w* is the width of the Gaussians from the previous stage. In effect, each Gaussian bead is kept in the vicinity of one Gaussian of the previous stage (with index *i* mod *n*). For the first stage, a zeroth stage with just one Gaussian placed at the origin was assumed as the previous one. The step size *d* was determined dynamically by slightly increasing or decreasing it after accepted or rejected steps, respectively.

The number of these hierarchical stages depended on the number of Gaussians in the final stage, which in turn depends on the desired resolution (as described above). Stages with fewer than 10 Gaussians did not substantially reduce the required number of annealing steps. Heuristically, the number of stages and the number of Gaussians per stage should therefore be chosen by repeatedly halving the final number of Gaussians until a value of about 10 is reached.

In each MCMC step, also a new common width σ of the Gaussians was proposed, with normally distributed proposals restricted to positive values. For the coliphage, also the heights *h_i_* of the Gaussians were determined. Here, separate MCMC steps with Gaussian proposals restricted to the uniform prior U(0.1,1) were performed for the heights alternatingly with those for the positions, with an independently determined step size.

The likelihood function from Eq. 2 is differentiable with respect to the electron density, such that it is possible to apply optimization methods that use gradient information, like, for example, stochastic gradient descent (SGD). While it is expected that such methods offer better scaling behavior (*51*), efficiently computing gradients on GPUs is challenging, in particular when noise is included in the forward model. In contrast, MCMC as described above is much more straightforward and was therefore used here to demonstrate the potential of our method. As a further benefit, MCMC allows to easily sample from the posterior, which is less straightforward using SGD.

### Regularized likelihood function

For stages of reduced resolution, a regularized version of the likelihood function was used. To that end, consider a smoothed version of the true electron density function ρ obtained by a convolution with a Gaussian kernel, $\tilde{\rho }=\rho *N\left(\sigma_{\text{reg}}\right)$. The intensity function corresponding to this smoothed version is, due to the Fourier convolution theorem, given by the pointwise product of the original intensity function and the squared absolute value of the Fourier transform of the smoothing kernel, $I_{\tilde{\rho }}\left(k\right)=I_{\rho }\left(k\right)\cdot \text{exp}\left(-\sigma_{\text{reg}}^{2}k^{2}\right)$. This relationship was used to obtain the images that would have been generated for the smoothed structure by rejection sampling, which were then used in the likelihood instead of the original images for the stages of reduced resolution.

In the noise-free case, computational efficiency was further increased substantially by selecting only those original images for the likelihood computations that actually contain useful information at the respective resolution. As described in the Supplementary Text, the Bayesian formalism allows for removing this selection bias.

### Structure alignment and resolution estimate

The resolution of the obtained electron densities was calculated using Fourier shell correlations (*48*).

$$\text{FSC}\left(k,\rho_{1},\rho_{2}\right)=\frac{\int_{∥k∥=k}{\hat{\rho }}_{1}{\left(k\right)}^{*}{\hat{\rho }}_{2}\left(k\right)\;dk}{\sqrt{\int_{∥k∥=k}{|{\hat{\rho }}_{1}\left(k\right)|}^{2}\;dk}\sqrt{\int_{∥k∥=k}{|{\hat{\rho }}_{2}\left(k\right)|}^{2}\;dk}}$$ (16)

where ρ_1_ and ρ_2_ are the densities to be compared and $\hat{\rho }$ denotes the Fourier transform of ρ. The achieved resolution was determined as 2π/*k*_fsc_(ρ_1_, ρ_2_), where *k*_fsc_(ρ_1_, ρ_2_) is the threshold at which the Fourier shell correlation drops below the conservative threshold of 0.5 (*48*). Because the orientations of the electron densities are random and irrelevant, they were aligned to each other before calculating the resolution, by maximizing *k*_fsc_(ρ_1_, **S**ρ_2_) over all orthogonal matrices **S** ∈ O(3). Both rotations and reflections were included, as x-ray scattering images do not distinguish between mirror images. Here, **S**ρ denotes the rotated electron density obtained by applying **S** to all positions **y***_i_* from Eq. 13.
