# Supplementary material for: Bayesian electron density determination from sparse and noisy single-molecule X-ray scattering images
Source: Sci Adv. 2024 Oct 25;10(43):eadp4425. doi: 10.1126/sciadv.adp4425 (PMC11506165; doi:10.1126/sciadv.adp4425)
Supplement: Supplementary file 1 — Supplementary Text Figs. S1 to S3 Table S1 Legend for movie S1 [file sciadv.adp4425_sm.pdf]

Science Advances
AAAS

# Supplementary Materials for

## Bayesian electron density determination from sparse and noisy single-molecule X-ray scattering images

Steffen Schultze and Helmut Grubmüller

Corresponding author: Helmut Grubmüller, hgrubmu@mpinat.mpg.de

*Sci. Adv.* **10**, eadp4425 (2024)
DOI: 10.1126/sciadv.adp4425

**The PDF file includes:**

Supplementary Text
Figs. S1 to S3
Table S1
Legend for movie S1

**Other Supplementary Material for this manuscript includes the following:**

Movie S1

# Supplementary Text

## Computation

We here describe the implementation of the likelihood computation for case of the noise-free model. The computations including noise and experimental effects are analogous, replacing, for example, the density of the Poisson distribution with that of the Gamma distribution. The source code is available at `https://gitlab.gwdg.de/sschult/xfel`.

The integral over SO(3) is approximated by a finite sum over rotations $\mathbf{R}_i$ with weights $w_i$,

$$P(\mathbf{k}_1, \ldots, \mathbf{k}_n \mid \rho) \approx \frac{N^n}{n!} \sum_i w_i \exp\left(-N \int_E I(\mathbf{R}_i \mathbf{k})\, \mathrm{d}\mathbf{k}\right) \prod_{j=1}^n I(\mathbf{R}_i \mathbf{k}_j) \tag{1}$$

Computing this sum involves evaluating the intensity function $I$ at all points of the form $\mathbf{R}_i\mathbf{k}_j$. Since this has to be done for all the images, this leads to a very large number of evaluations of $I$. By discretizing the photon positions $\mathbf{k}_j$ and choosing a suitable set of orientations $R_j$ it can be ensured that many of these points $\mathbf{R}_i\mathbf{k}_j$ coincide, which greatly increases the computational efficiency.

To construct this set of orientations, we proceed as follows. First, we choose a Lebedev grid as a uniform grid of points $\mathbf{v}_i$ in the 2-sphere $S^2$. For each one of these, we find a rotation $Q_i \in \mathrm{SO}(3)$ such that $Q_i\mathbf{v}_i \parallel \mathbf{k}_0$. In addition, let $S_j$ be uniformly spaced rotations around the axis defined by $\mathbf{k}_0$. The set of products $S_jQ_i$ is then a uniform grid in SO(3). The Lebedev precision and the number of angular rotations $S_j$ were chosen such that the expected angular distance between nearest neighbors in the resulting grid is smaller than the length scale corresponding to the desired relative resolution divided by the approximate radius of the molecule.

For the discretization, a radially symmetric grid of cells with centers $\mathbf{x}_{k,l}$ and areas $a_{k,l}$ is chosen, such that $S_j\mathbf{x}_{k,l} = \mathbf{x}_{k+j,l}$. Here, the first index is considered cyclic, that is, if, say, $k$ ranges from 1 to $k_{\max}$, then $\mathbf{x}_{k+j,l}$ is to be interpreted as $\mathbf{x}_{(k+j \mod k_{\max}),l}$. Because the corresponding areas $a_{k,l}$ only depend on $l$, we write $a_l = a_{k,l}$ as a shorthand. Each image is now described by the indices $k_1, l_1, \ldots, k_n, l_n$ of the cells $\mathbf{x}_{k,l}$ that are closest to the photon positions $\mathbf{k}_1, \ldots, \mathbf{k}_n$.

Plugging in these definitions, the likelihood now reads

$$P(k_1, l_1, \ldots, k_n, l_n \mid \rho) \approx \frac{N^n}{n!} \sum_{i,j} w_i \exp\left(-N \sum_{k,l} a_l I(Q_iS_j\mathbf{x}_{k,l})\right) \prod_{m=1}^n a_{l_m} I(Q_iS_j\mathbf{x}_{k_m,l_m}) \tag{2}$$

$$= \frac{N^n}{n!} \sum_i w_i \exp\left(-N \sum_{k,l} a_l I(Q_i\mathbf{x}_{k,l})\right) \sum_j \prod_{m=1}^n a_{l_m} I(Q_i\mathbf{x}_{k_m+j,l_m}) \tag{3}$$

$$= \frac{N^n}{n!} \sum_i w_i P_i \sum_j \prod_{m=1}^n I_{i,k_m+j,l_m} \tag{4}$$

The values $I_{i,k,l} \coloneqq a_l I(Q_i\mathbf{x}_{k,l})$ and $P_i \coloneqq \exp(-N \sum_{k,l} I_{i,k,l})$ are computed in advance and reused for each image.

Due to limited floating point precision, a number of adjustments must be made. Due to the large value of $N$, computing $P_i$ results in underflow. Therefore, we write

$$\tilde{P}_i = P_i/\bar{P}, \qquad \bar{P} = \left(\prod_{i'=1}^{i_{\max}} P_{i'}\right)^{\frac{1}{i_{\max}}}. \tag{5}$$

Further, $I_{i,k,l} \ll 1$, so if the images contain enough photons the product over $m$ will underflow. Since the magnitude of $I_{i,k,l}$ depends mostly on $l$, we define

$$\tilde{I}_{i,k,l} = I_{i,k,l}/\bar{I}_l, \qquad \bar{I}_l = \frac{1}{i_{\max} k_{\max}} \sum_{i'=1}^{i_{\max}} \sum_{k'=1}^{k_{\max}} I_{i',k',l} \tag{6}$$

Both $\bar{P}$ and $\bar{I}_l$ do not depend on the rotation index $i$ and factor out,

$$P(k_1, l_1, \ldots, k_n, l_n \,|\, \rho) \approx \frac{N^n}{n!} \bar{P} \left(\prod_{m=1}^{n} \bar{I}_{l_m}\right) \sum_i w_i \tilde{P}_i \sum_j \prod_{m=1}^{n} \tilde{I}_{i,k_m+j,l_m} \tag{7}$$

Taking the logarithm,

$$\log P(k_1, l_1, \ldots, k_n, l_n \,|\, \rho) \approx \log \frac{N^n}{n!} + \log \bar{P} + \sum_{m=1}^{n} \log \bar{I}_{l_m} + \log \sum_i w_i \tilde{P}_i \sum_j \prod_{m=1}^{n} \tilde{I}_{i,k_m+j,l_m}, \tag{8}$$

we see that only $\log \bar{P}$ and $\log \bar{I}_l$ appear, which can be computed without overflow.

## Image selection

In our hierarchical sampling scheme, images containing only photons with $k$ below a threshold are no longer useful, and the computations were sped up by removing these images. To achieve this, only the subset $\mathcal{I}_C$ of those images was used that fulfilled the condition $C(I)$ that for each $i$ the image $I$ contains at least $m_i$ photons with $r_i < k < r_{i+1}$, where the parameters $r_i$ and $m_i$ were chosen such that the radial distribution of photons in the selected images was close to uniform up to the desired resolution level (Table S1). To ensure that the posterior was not biased by this filtering, it was taken into account in the Bayesian formalism by dividing by the probability $P(C \,|\, \rho)$ that an image fulfills $C$. In other words, the original posterior probability was replaced with $P(\rho \,|\, \mathcal{I}_C, C) \propto P(\mathcal{I}_C \,|\, \rho)/P(C \,|\, \rho)$. Because the probability that an image fulfills $C$ depends on the orientation $\mathbf{R}$, $P(C \,|\, \rho)$ was obtained by averaging over $\mathbf{R}$,

$$P(C \,|\, \rho) = \int_{\mathrm{SO}(3)} \prod_i \left(1 - Q\left(m_i - 1, N \int_{E_i} I(\mathbf{R}_i \mathbf{k})\, \mathrm{d}\mathbf{k}\right)\right) \mathrm{d}\mathbf{R}, \tag{9}$$

where $Q(x, \lambda)$ is the cumulative distribution function of a Poisson distribution with mean $\lambda$ and $E_i = \{\mathbf{k} \in E \,|\, r_i < \|\mathbf{k}\| < r_{i+1}\}$ are the relevant slices of the Ewald sphere. This integral over $\mathbf{R}$ was implemented by the same quadrature rule as described above.

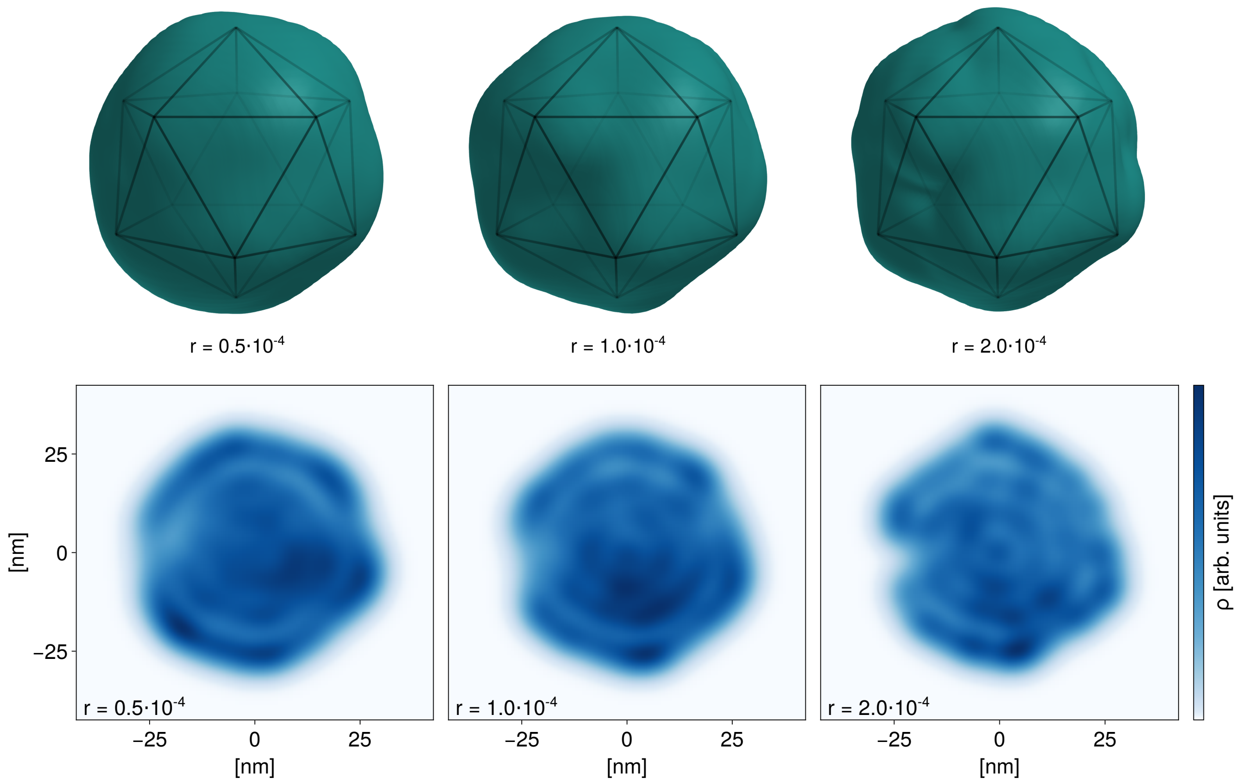


**Figure S1. Dependence of determined electron densities on downsampling ratio.** Average electron density obtained for the coliphage PR772 at three downsampling ratios $r$, shown as isosurfaces (top) and slices (bottom).

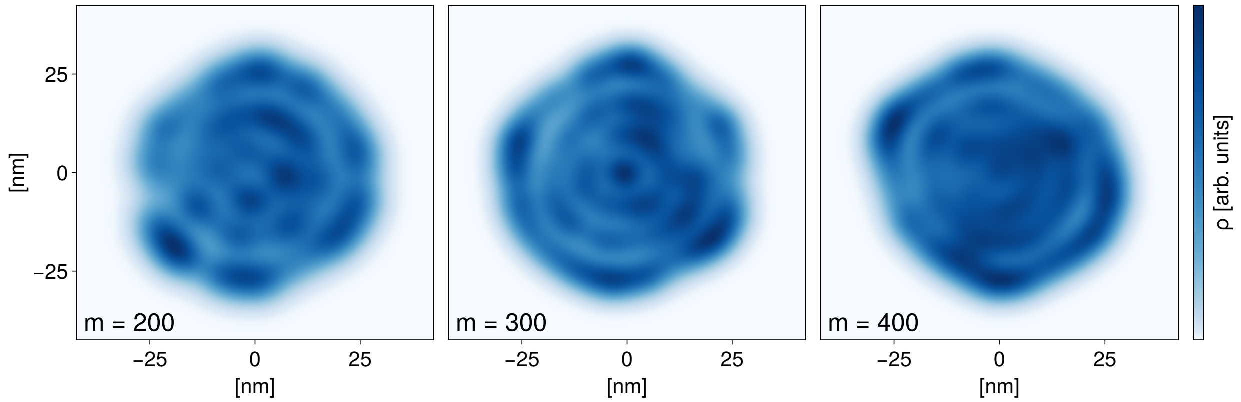


**Figure S2. Electron density determination using different numbers of Gaussians.** Average electron densities obtained for the coliphage PR772 using representations of $m = 200$, $m = 300$ and $m = 400$ Gaussians, at a downsampling ratio $r = 10^{-4}$.

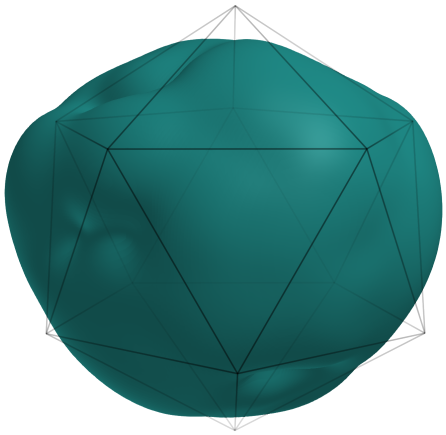

**Figure S3. Effect of using the 'wrong' likelihood function.** Average electron density obtained using the 'wrong' likelihood function (eq. 11 instead of eq. 12) for the coliphage data set.

| Test Case | Stage | total images | selected images | $n_i$ | $r_i$ [1/Å] | $\sigma_{\mathrm{reg}}$ [Å] | $t_{1/2}$ | number of Gaussians | Lebedev precision | angular rotations |
|---|---|---|---|---|---|---|---|---|---|---|
| crambin noise-free | 1 | $8.96 \cdot 10^3$ | 1,000 | 4 | $0.25, \infty$ | 2.0 | $1 \cdot 10^3$ | 12 | 23 | 32 |
| | 2 | $1.00 \cdot 10^7$ | 19,315 | 3, 2 | $0.33, 0.5, \infty$ | 1.5 | $1 \cdot 10^4$ | 23 | 47 | 32 |
| | 3 | $3.04 \cdot 10^6$ | 50,000 | 1, 1, 2 | $0.35, 0.5, 0.65, \infty$ | 1.2 | $2 \cdot 10^4$ | 46 | 47 | 64 |
| | 4 | $1.00 \cdot 10^8$ | 204,447 | 1, 2, 2 | $0.35, 0.5, 0.8, \infty$ | 0.9 | $1 \cdot 10^5$ | 92 | 89 | 64 |
| | 5 | $1.00 \cdot 10^8$ | 634,032 | 1, 1, 3 | $0.4, 0.65, 0.9, \infty$ | 0.5 | $1 \cdot 10^5$ | 184 | 89 | 64 |
| crambin 75 % noise | 1 | $1.00 \cdot 10^6$ | - | - | - | 1.0 | $5 \cdot 10^2$ | 12 | 23 | 32 |
| | 2 | $1.00 \cdot 10^6$ | - | - | - | 0.0 | $1 \cdot 10^4$ | 23 | 47 | 64 |
| crambin 90 % noise | 1 | $3.00 \cdot 10^6$ | - | - | - | 1.0 | $5 \cdot 10^2$ | 12 | 23 | 32 |
| Coliphage PR772 | 1 | $1.50 \cdot 10^3$ | - | - | - | 0.0 | $1 \cdot 10^4$ | 13 | 23 | 64 |
| | 2 | $2.00 \cdot 10^5$ | - | - | - | 0.0 | $5 \cdot 10^4$ | 400 | 35 | 64 |

**Table S1**
**Parameters used for each of the test cases.**

**Movie S1. Determined average electron density of coliphage PR772.** Rotating 3D isosurface of the averaged determined electron density for coliphage PR772. Perfect icosahedron of side length 30 nm for reference. Isosurface drawn at 5 % of the maximum value of $\rho$.
